# Pharmacokinetics and dialytic clearance of apixaban during in vitro continuous renal replacement therapy

**DOI:** 10.1186/s12882-021-02248-7

**Published:** 2021-01-30

**Authors:** Lauren Andrews, Scott Benken, Xing Tan, Eric Wenzler

**Affiliations:** College of Pharmacy, University of Illinois at Chicago, 833 South Wood Street, Room 164 (M/C 886),, Chicago, IL 60612 USA

**Keywords:** Apixaban, Pharmacokinetics, Dialysis, Renal replacement therapy, Sieving coefficient, Saturation coefficient, Transmembrane clearance, CRRT, CVVH, CVVHD

## Abstract

**Background:**

To evaluate the transmembrane clearance (CL_TM_) of apixaban during modeled in vitro continuous renal replacement therapy (CRRT), assess protein binding and circuit adsorption, and provide initial dosing recommendations.

**Methods:**

Apixaban was added to the CRRT circuit and serial pre-filter bovine blood samples were collected along with post-filter blood and effluent samples. All experiments were performed in duplicate using continuous veno-venous hemofiltration (CVVH) and hemodialysis (CVVHD) modes, with varying filter types, flow rates, and point of CVVH replacement fluid dilution. Concentrations of apixaban and urea were quantified via liquid chromatography-tandem mass spectrometry. Plasma pharmacokinetic parameters for apixaban were estimated via noncompartmental analysis. CL_TM_ was calculated via the estimated area under the curve (AUC) and by the product of the sieving/saturation coefficient (SC/SA) and flow rate. Two and three-way analysis of variance (ANOVA) models were built to assess the effects of mode, filter type, flow rate, and point of dilution on CL_TM_ by each method. Optimal doses were suggested by matching the AUC observed in vitro to the systemic exposure demonstrated in Phase 2/3 studies of apixaban. Linear regression was utilized to provide dosing estimations for flow rates from 0.5–5 L/h.

**Results:**

Mean adsorption to the HF1400 and M150 filters differed significantly at 38 and 13%, respectively, while mean (± standard deviation, SD) percent protein binding was 70.81 ± 0.01%. Effect of CVVH point of dilution did not differ across filter types, although CL_TM_ was consistently significantly higher during CRRT with the HF1400 filter compared to the M150. The three-way ANOVA demonstrated improved fit when CL_TM_ values calculated by AUC were used (adjusted R^2^ 0.87 vs. 0.52), and therefore, these values were used to generate optimal dosing recommendations. Linear regression revealed significant effects of filter type and flow rate on CL_TM_ by AUC, suggesting doses of 2.5–7.5 mg twice daily (BID) may be needed for flow rates ranging from 0.5–5 L/h, respectively.

**Conclusion:**

For CRRT flow rates most commonly employed in clinical practice, the standard labeled 5 mg BID dose of apixaban is predicted to achieve target systemic exposure thresholds. The safety and efficacy of these proposed dosing regimens warrants further investigation in clinical studies.

**Supplementary Information:**

The online version contains supplementary material available at 10.1186/s12882-021-02248-7.

## Background

Venous thromboembolism (VTE) and new-onset atrial fibrillation (NOAF) occur in up to 37 and 46% of critically ill patients, respectively, and are responsible for significant morbidity and mortality in the intensive care unit (ICU) [1–7]. Parenteral anticoagulation with unfractionated heparin (UFH) is the current mainstay therapy for VTE and NOAF in this population given its rapid onset of action and short half-life [8]. Due to the potential for serious adverse effects with UFH, activated thromboplastin time (aPTT) monitoring and dose titration are required to maintain exposures within a narrow therapeutic index [9]. Despite the assistance of dosing algorithms and decades of clinical experience, > 75% of hospitalized patients fail to achieve aPTTs within goal range during the first 24–48 h after UFH initiation and only 29% are able to sustain them [10–12]. In the critically ill ICU population, only approximately 50% of patients achieve therapeutic aPTTs within this same time frame, largely secondary to pathophysiologic and pharmacokinetic (PK) derangements such as sepsis-induced acute kidney injury (AKI), which can necessitate renal replacement therapy (RRT) in up to 70% of patients [13–21]. Alternatively, despite minimal PK alterations, low molecular weight heparin (LMWH) has demonstrated detrimental pharmacodynamic (PD) properties, including increased thrombin generation time and an increased risk of bleeding in patients receiving RRT [22–25]. Given the challenges in optimizing the use of UFH and LMWH in the ICU setting, more reliable anticoagulation therapies are desperately needed for managing critically ill patients, especially those requiring extracorporeal organ support such as continuous RRT (CRRT).

Apixaban, a direct-acting oral anticoagulant (DOAC) agent, has emerged as a potential alternative therapy for the treatment of VTE and NOAF over UFH and LMWH in the ICU population due to its more reliable dose-exposure-response relationship, lack of required monitoring, decreased drug-drug interactions, and improved safety profile [8, 26–31]. Pharmacokinetic studies in otherwise healthy subjects with end stage renal disease (ESRD) on intermittent hemodialysis (HD) indicate that HD has a limited overall impact on the clearance (CL) of apixaban [32–35]. However, data regarding CL by conventional HD cannot be accurately extrapolated to CRRT given the differences in modes, durations of therapy, types of hemofilters used, and blood, ultrafiltration, and dialysate flow rates [36]. Unfortunately, robust PK data in patients receiving CRRT are scarce and often include only small numbers of critically ill patients on many different forms of CRRT with heterogeneous flow rates, filter types, dosing, and sampling schemes, making it difficult to draw meaningful conclusions [37–41]. As such, in vitro CRRT models are useful for generating precise assessments of sieving/saturation coefficients (SC/SA) across different modes, flow rates, filter types, and points of dilution while eliminating the variability introduced by the patient. As recognized by the U. S Food and Drug Administration (FDA), National Institutes of Health (NIH), and National Institute of Allergy and Infectious Diseases (NIAID), these models can be used to guide dosing in the absence of, or when combined with, in vivo data and have been shown to accurately predict in vivo total body clearance (CL_T_) [42], allowing for data derived from in vitro investigations to be utilized in estimating clinical dosing regimens [43].

Although apixaban may represent a safer and more efficacious alternative to UFH and LMWH in ICU patients, the lack of PK data to inform appropriate dosing in patients undergoing CRRT currently precludes its use in a significant proportion of the critically ill population. As such, the objective of this study was to evaluate the PK and dialytic clearance of apixaban during in vitro CRRT in order to provide initial guidance on optimal dosing in this population.

## Methods

### Study design

#### In vitro CRRT clearance model

In vitro CRRT was simulated using a Prismaflex 7.2 control unit (Baxter Healthcare Corporation, Deerfield, IL, USA) in continuous veno-venous hemofiltration (CVVH) and continuous veno-venous hemodialysis (CVVHD) modes using fresh 1.4m^2^ polyarylethersulfone (PAES; Prismaflex HF1400) and 1.5m^2^ acrylonitrile (AN69; Prismaflex M150) hemofilter sets for each experiment. One liter of heparinized (20 units/mL) whole bovine blood (Densco Marketing Inc., Woodstock, IL, USA) was heated to 37 °C in a water bath and stirred continuously. The Prismaflex circuit was initially primed with 186 mL (HF1400) or 189 mL (M150) of 0.9% sodium chloride per the manufacturer’s operating instructions [44, 45]. Prior to the start of each experiment, blood was then allowed to circulate throughout the system for at least 2 min to permit adequate exposure of the hemofilter to blood proteins. The blood flow rate was fixed at 200 mL/min for all experiments while CVVH replacement fluid (PrismaSOL® BGK 2/0; Baxter Healthcare Corporation, Deerfield, IL, USA) and CVVHD dialysate (PrismaSATE® BGK 2/0; Baxter Healthcare Corporation, Deerfield, IL, USA) rates of 2 L/h and 4 L/h were tested with each filter type. During CVVH at 2 L/h, replacement fluid was added 100% pre-filter, 100% post-filter, and at 50% pre−/50% post-filter. During CVVH at 4 L/h, replacement fluid was added at 50% pre−/50% post-filter. All experiments were performed in duplicate in each mode, at each rate, and with each filter for a total of 24 experiments (excluding adsorption experiments).

Apixaban (Eliquis®; Bristol-Myers Squibb, New York, NY, USA) was reconstituted per manufacturer’s instructions [46]. To account for measured bovine hematocrit of 36.9% (Biologic Resources Laboratory, University of Illinois at Chicago, Chicago, IL, USA), the dose of apixaban added to the central reservoir was adjusted a priori to simulate the mean peak serum concentration observed in healthy adult subjects following a single 5 mg dose of apixaban (~ 0.104 mg/L) [47]. Urea (Sigma-Aldrich, St. Louis, MO, USA) was also added at 75 mg/L to serve as the control solute.

After at least 1 min of equilibration, serial pre-filter blood samples were collected in 3.2% sodium citrate tubes (Becton, Dickinson and Company, Franklin Lakes, NJ, USA) at 0, 10, 20, 30, 45, and 60 min post-dose with analogous post-filter blood and effluent samples collected at 10 and 30 min. Blood samples were centrifuged at 1500 x *g* for 10 min and the resultant supernatant plasma and ultrafiltrate samples were frozen at − 80 °C within 30 min of collection until analysis.

#### Adsorption experiments

To evaluate potential adsorption of apixaban to the hemofilters, the initial CRRT model was modified to create a closed-circuit system. Effluent was rerouted to the central blood reservoir, and 0.9% normal saline was exogenously pumped into the effluent bag via a Masterflex® Peristaltic pump (Cole-Parmer, Vernon Hills, IL, USA) at the same rate to prevent the Prismaflex system from aborting due to the patient blood loss/gain alarm. Serial blood samples were drawn from the central reservoir at 0, 10, 20, 30, 45, 60, 90, 105, 120, 150, and 180 min, centrifuged at 1500 *x g* for 10 min, and supernatant plasma was frozen at − 80 °C within 30 min of collection until analysis. This process was repeated twice in duplicate for a total of 4 experiments.

#### Protein binding determination

To assess apixaban protein binding in bovine plasma, 4 contrived samples were centrifuged at 2000 x *g* for 30 min using a Centrifree® Ultrafiltration Device (Merck Millipore Ltd. Tullagreen, Carrigtwohill, Co. Cork, Ireland) with resulting bound and unbound plasma samples frozen at − 80 °C within 30 min until analysis. This process was repeated twice in duplicate for a total of 4 experiments.

#### Bioanalytical procedures

Concentrations of apixaban and urea in bovine plasma and effluent were quantified via liquid chromatography-tandem mass spectrometry (Keystone Bioanalytical, North Wales, PA, USA) as previously described [48]. The calibration range of the assay was linear from 0.001–0.2 mg/L (*r* ≥ 0.999). The precision and accuracy acceptance criteria for the quality control (QC) samples and calibration standards were ≤ 15% coefficient of variance (CV) and ± 15% relative error (RE) determined at each concentration level.

#### Pharmacokinetic procedures

Pharmacokinetic parameters for apixaban were estimated from observed pre-filter plasma concentrations via noncompartmental analysis in Phoenix WinNonlin Version 8.1 (Certara USA Inc., Princeton, NJ, USA). Reported parameters included: maximum plasma concentration (C_max_), last observed plasma concentration (C_last_), elimination rate constant (K_e_), half-life (t_1/2_), apparent volume of distribution (V_d_), clearance (CL), and the area under the concentration-time curve (AUC_0-∞_ and AUC_0-last_) as determined via the linear up-log down method. As in vitro experiments were performed over a period of one hour, AUC_0-last_ was multiplied by 24 to demonstrate proportional AUC_0–24_. Calculations for the estimation of apixaban and urea removal from the CRRT circuit were as follows:

$$ \mathrm{sieving}\ \mathrm{coefficient}\ \left(\mathrm{SC}\right)=\left({\mathrm{C}}_{\mathrm{uf}}/{\mathrm{C}}_{\mathrm{pre}}\right) $$

$$ \mathrm{saturation}\ \mathrm{coefficient}\ \left(\mathrm{SA}\right)=\left(2\ast {\mathrm{C}}_{\mathrm{dialysate}}\right)/\left({\mathrm{C}}_{\mathrm{pre}}+{\mathrm{C}}_{\mathrm{post}}\right) $$

Where C_uf_ is the concentration in the ultrafiltrate, C_pre_ is the concentration from the pre-filter sampling port, C_dialysate_ is the concentration in the dialysate, and C_post_ is the concentration from the post-filter sampling port [49–51].

Clearance by CRRT was then estimated by two distinct methods to ensure accuracy and allow for comparison. The primary method of estimating CL_TM_ was estimated using the AUC_0–24_ determined via noncompartmental analysis (CL_TM_ by AUC), as previously described. The secondary method, CL_TM_ by sieving/ saturation coefficients (SC/SA), utilized the following equations:

$$ {\mathrm{CL}}_{\mathrm{CVVH}}=\left(\mathrm{SC}\ast {\mathrm{Q}}_{\mathrm{uf}}\ast {\mathrm{Q}}_{\mathrm{b}}\right)/\left({\mathrm{Q}}_{\mathrm{b}}+{\mathrm{Q}}_{\mathrm{rep}}\right) $$

$$ {\mathrm{CL}}_{\mathrm{CVVHD}}=\left(\mathrm{SA}\ast {\mathrm{Q}}_{\mathrm{d}}\right) $$

Where Q_uf_ is the ultrafiltrate or replacement fluid flow rate and Q_d_ is the dialysate flow rate. In experiments where replacement fluid was added pre-filter, a dilutional correction factor was incorporated into the clearance equation, with Q_b_ representing blood flow rate and Q_rep_ being the pre-filter replacement fluid rate [51, 52].

Adsorption was calculated as the difference between the total amount of apixaban added to the system and the total amount recovered in the dialysate and plasma after 180 min using the following equation at each sampling time point [53]:

$$ {\displaystyle \begin{array}{l}\mathrm{Adsorption}\ \left(\%\right)=\Sigma 1-\left[\left(\mathrm{dose}\ \mathrm{of}\ \mathrm{apixaban}\ \mathrm{added}\ \mathrm{at}\ \mathrm{time}\ \mathrm{zero}\right)/\right(\mathrm{concentration}\ \mathrm{of}\\ {}\mathrm{apixaban}\ast \mathrm{measured}\ \mathrm{volume}\ \mathrm{in}\ \mathrm{central}\ \mathrm{reservoir}\left)\right]\end{array}} $$

#### Optimal dosing determination

Optimal dosing was calculated to provide a comparable mean AUC value to that achieved following the administration of apixaban 5 mg twice daily for 7 days in healthy subjects (2103.8 mg · h/L) via the equation AUC = Dose / CL_T_; where CL_T_ = CL_TM_ + CL_NR_ [54]. Here, CL_T_ represents total body clearance, CL_TM_ represents CL via CRRT, and CL_NR_ is non-renal clearance. The value for CL_NR_ (2.52 L/h) was imputed from Phase 2 and 3 studies evaluating apixaban for the treatment or prevention of recurrent VTE and assumed to be constant [55, 56]. Additionally, residual renal function was assumed to be negligible as the majority of critically ill patients with AKI on CRRT have no appreciable residual renal function [52].

### Statistical analysis

Data are presented as mean (± standard deviation, SD) or with 95% confidence intervals (95% CI). Continuous data were compared via Student’s t-test. Additionally, one- and two-way analysois of variance (ANOVA) models with Tukey’s post-hoc tests were built to evaluate statistically significant differences in mean CL_TM_ according to CVVH point of dilution within and between each filter type, respectively. Then, three-way ANOVA models were fit using CL_TM_ as the outcome to evaluate the interaction between CRRT mode, filter type, and flow rate. ANOVA-generated means and 95% CI of CL_TM_ were then used to estimate AUC and optimal total daily doses (TDD) of apixaban during CRRT. Finally, multiple linear regression via backwards stepwise analysis was used to correlate flow rate with mean CL_TM_ while adjusting for covariates (CRRT mode, filter type, point of dilution, and flow rate) and predict optimal dosing regimens across flow rates from 0.5–5 L/h. Model performance was assessed via the adjusted R^2^ value. Collinearity was assessed via tolerance and variance inflation factor. A *P* value of ≤0.05 was considered statistically significant in the final model. All statistical analyses were performed using SPSS® Version 26 (IBM Corp, Armonk, NY, USA).

## Results

### In vitro CRRT clearance model

**Fig. 1:**
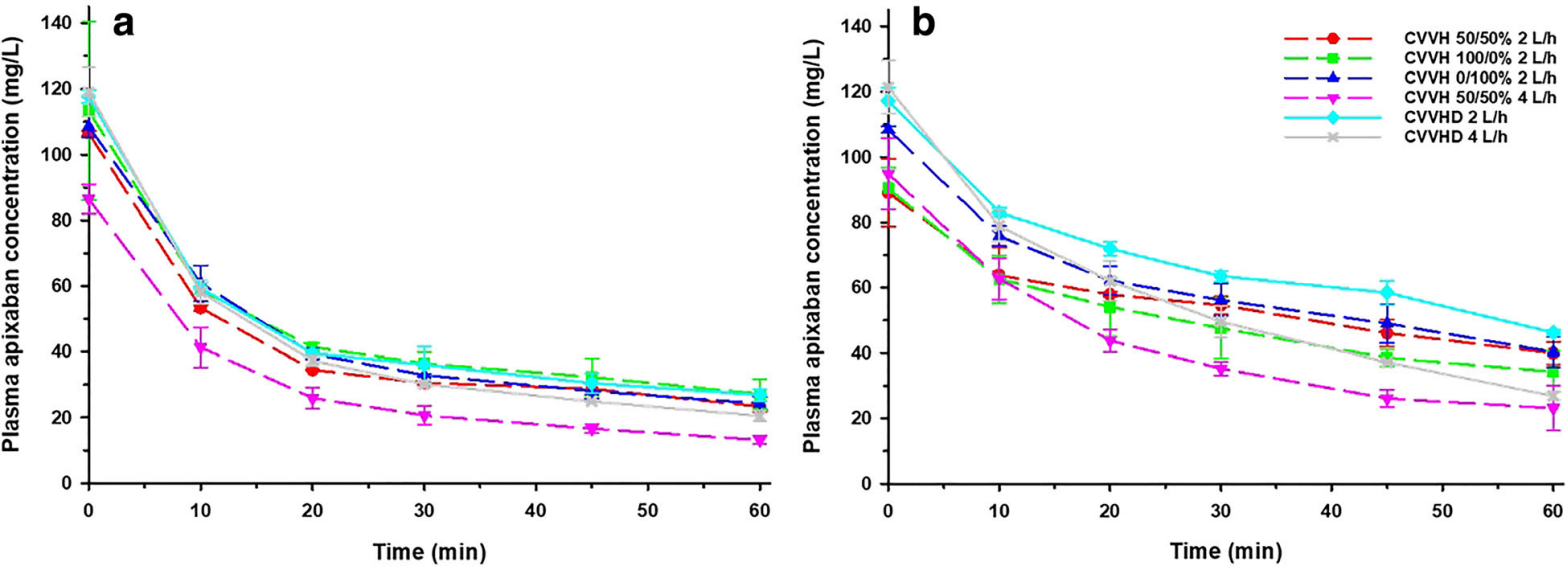
Pre-filter plasma concentration-time profiles of apixaban during CVVH and CVVHD at each rate and point of dilution with the HF1400 filter (**a**) and M150 filter (**b**). Mean values are displayed with error bars representing standard deviations

Mean (±SD) pre-filter PK parameters of apixaban in bovine plasma during CRRT as estimated via noncompartmental analyses are summarized in Table 1, and respective plasma concentration-time profiles are shown in Fig. 1. The mean (±SD) C_max_ value observed across the 24 experiments was 0.106 ± 0.014 mg/L (< 2% difference from target value of 0.104 mg/L). Notably, CL_TM_ by AUC did not scale proportionally with flow rate, with increases in CL_TM_ ranging from 1.3–1.8-fold as flow rate increased from 2 to 4 L/h.

**Table 1:** Bovine plasma pharmacokinetic parameters of apixaban during in vitro CRRT as determined via noncompartmental analyses^a^

	C_max_ (ng/mL)	C_last_ (ng/mL)	K_e_ (hr^−1^)	*t*_1/2_ (h)	V_d_ (L)	CL_TM_ (L/h)	AUC_0-∞_ (ng · h/mL)	AUC_0-last_ (ng · h/mL)	AUC_0–24_ (ng · h/mL)
CVVH					HF1400				
2 L/h, 50/50%	106.5 ± 0.7	23.4 ± 1.6	0.9 ± 0.0	0.8 ± 0.0	2.2 ± 0.0	1.9 ± 0.1	66.8 ± 2.7	39.3 ± 0.2	943.6 ± 4.9
2 L/h, 100/0%	113.3 ± 21.2	21.2 ± 4.5	0.9 ± 0.2	0.8 ± 0.2	2.0 ± 0.0	1.7 ± 0.4	78.2 ± 18.5	44.5 ± 5.0	1068.9 ± 120.5
2 L/h, 0/100%	108.5 ± 3.2	24.3 ± 2.0	1.0 ± 0.2	0.7 ± 0.2	1.9 ± 0.2	1.9 ± 0.2	66.9 ± 7.2	42.1 ± 0.3	1009.8 ± 6.8
4 L/h, 50/50%	86.5 ± 4.5	13.1 ± 1.2	1.3 ± 0.1	0.5 ± 0.0	2.6 ± 0.4	3.3 ± 0.3	38.2 ± 3.4	27.9 ± 3.1	669.4 ± 73.5
CVVHD									
2 L/h	117.7 ± 1.9	26.9 ± 1.8	0.9 ± 0.1	0.8 ± 0.1	1.9 ± 0.0	1.7 ± 0.2	75.7 ± 8.1	44.1 ± 2.2	1058.0 ± 52.2
4 L/h	119.1 ± 7.4	20.5 ± 1.6	1.2 ± 0.2	0.6 ± 0.1	1.9 ± 0.2	2.2 ± 0.1	58.1 ± 3.5	40.1 ± 0.4	962.7 ± 9.7
CVVH					M150				
2 L/h, 50/50%	107.9 ± 16.2	31.0 ± 10.2	0.9 ± 0.3	0.9 ± 0.3	1.8 ± 0.4	1.6 ± 0.7	92.5 ± 37.0	49.6 ± 11.8	1191.0 ± 283.3
2 L/h, 100/0%	90.4 ± 13.8	34.3 ± 9.9	0.7 ± 0.3	0.9 ± 0.2	1.8 ± 0.3	1.3 ± 0.6	97.1 ± 25.6	50.6 ± 10.8	1214.3 ± 259.3
2 L/h, 0/100%	108.4 ± 13.3	40.3 ± 9.5	0.7 ± 0.3	1.0 ± 0.2	1.5 ± 0.3	1.1 ± 0.6	117.6 ± 28.6	60.8 ± 10.7	1458.6 ± 256.9
4 L/h, 50/50%	94.9 ± 15.1	23.2 ± 9.9	1.2 ± 0.3	0.6 ± 0.2	1.7 ± 0.4	2.1 ± 0.7	62.2 ± 29.9	42.1 ± 11.8	1009.3 ± 283.1
CVVHD									
2 L/h	117.3 ± 14.2	46.2 ± 10.4	0.7 ± 0.2	1.1 ± 0.2	1.4 ± 0.4	0.9 ± 0.7	139.2 ± 32.6	69.0 ± 12.8	1656.1 ± 307.5
4 L/h	121.4 ± 14.3	26.9 ± 9.7	1.3 ± 0.3	0.5 ± 0.2	1.3 ± 0.3	1.6 ± 0.6	77.2 ± 29.3	56.1 ± 11.2	1346.3 ± 269.0

Data are presented as mean ± SD

^a^n = 2 experiments in each mode, at each flow rate, point of dilution, and filter type (24 total experiments)

Table 2 displays the observed SC, SA, and CL_TM_ by SC/SA values of apixaban and urea during CVVH and CVVHD stratified by CRRT mode, filter type, flow rate, and point of replacement fluid dilution. As expected, CL_TM_ by SC/SA values scaled roughly proportional to increases in flow rate from 2 to 4 L/h during CVVH (1.86–2.34 L/h) and CVVHD (1.94 L/h). The mean apixaban SC value during CVVH with the M150 filter was 0.584 and 0.594 with HF1400 (*P* = 0.726) and mean SA during CVVHD was 0.587 with M150 and 0.612 with HF1400 (*P* = 0.543). The average SC across CVVH was 0.589 and SA across CVVHD was 0.599 (*P* = 0.628). The overall mean SC/SA for apixaban was 0.594 across all CRRT modes, filter types, flow rates, and points of replacement fluid dilution tested, while overall mean CL_TM_ by SC/SA at 2 and 4 L/h were 1.2 and 2.4 L/h, respectively. Urea SC, SA, and CL_TM_ values were comparable to previously established parameters obtained in analogous experimental conditions [57].

**Table 2:** Transmembrane clearance of apixaban and urea during in vitro CRRT as determined by SC or SA * flow rate^a^

	Apixaban		Urea	
	SC	CL_TM_ (L/h)	SC	CL_TM_ (L/h)
CVVH	HF1400			
2 L/h, 50/50%	0.608 ± 0.08	1.22 ± 0.16	1.00 ± 0.03	2.00 ± 0.06
2 L/h, 100/0%	0.531 ± 0.06	1.06 ± 0.11	0.99 ± 0.08	1.98 ± 0.15
2 L/h, 0/100%	0.614 ± 0.03	1.23 ± 0.06	1.09 ± 0.03	2.17 ± 0.06
4 L/h, 50/50%	0.622 ± 0.04	2.49 ± 0.17	1.12 ± 0.03	4.47 ± 0.11
CVVHD	SA	CL_TM_ (L/h)	SA	CL_TM_ (L/h)
2 L/h	0.622 ± 0.12	1.24 ± 0.24	1.23 ± 0.08	2.46 ± 0.16
4 L/h	0.601 ± 0.11	2.40 ± 0.43	1.38 ± 0.17	5.53 ± 0.67
CVVH	M150			
2 L/h, 50/50%	0.612 ± 0.03	1.22 ± 0.06	1.08 ± 0.10	2.15 ± 0.19
2 L/h, 100/0%	0.581 ± 0.07	1.16 ± 0.14	1.01 ± 0.07	2.02 ± 0.14
2 L/h, 0/100%	0.603 ± 0.02	1.21 ± 0.04	1.08 ± 0.05	2.16 ± 0.09
4 L/h, 50/50%	0.540 ± 0.04	2.16 ± 0.17	1.00 ± 0.07	3.98 ± 0.29
CVVHD	SA	CL_TM_ (L/h)	SA	CL_TM_ (L/h)
2 L/h	0.619 ± 0.01	1.24 ± 0.03	1.19 ± 0.05	2.38 ± 0.10
4 L/h	0.555 ± 0.05	2.22 ± 0.20	1.27 ± 0.17	5.09 ± 0.66

Data are presented as mean ± SD

^a^n = 2 experiments in each mode, at each flow rate, point of dilution, and filter type (24 total experiments)

### Effect of CVVH point of dilution

Within the HF1400 filter group, there were no significant differences in CL_TM_ by AUC across any of the 3 points of dilution at 2 L/h (*P* = 0.641), however, when evaluated as CL_TM_ by SC, there were significant differences noted between the 50/50% and both the 0/100% (*P* = 0.009) and 100/0% modes (*P* = 0.014). Within the M150 group, point of replacement fluid addition also did not affect CL_TM_ by AUC (*P* = 0.420), but again demonstrated significant differences between the 50/50% and both the 0/100% (*P* = 0.003) and 100/0% (*P* = 0.004) groups by SC.

### Effect of filter type

At 2 L/h, filter type did significantly affect CL_TM_ by AUC where the mean CL_TM_ was 1.80 L/h (95% CI 1.56–2.05) for the HF1400 filter vs 1.12 L/h (95% CI 0.87–1.37) for the M150 filter (*P* = 0.001). No difference was observed at 4 L/h: 3.30 L/h (95%CI 0.63–5.97) for HF1400 and 2.07 L/h (95%CI -2.12-6.26) for M150 (*P* = 0.088), although there were only 2 experiments per group at this rate. Including both flow rates, mean CL_TM_ values by AUC differed significantly between filters (HF1400 2.18 L/h, 95% CI 1.57–2.78 vs. M150 1.36 L/h, 95% CI 0.93–1.79, *P* = 0.021).

For CL_TM_ by SC/SA at 2 L/h, filter type did not have a significant effect with mean CL_TM_ values of 1.44 L/h (95% CI 0.97–1.91) for HF1400 and 1.51 L/h (95% CI 0.97–2.04) for M150 (*P* = 0.810). There was also no difference observed at 4 L/h: 2.49 L/h (95% CI 0.58–4.39) for HF1400 and 2.16 L/h (95% CI 0.89–3.43) for M150 (*P* = 0.209). Including both flow rates, mean CL_TM_ values by SC/SA remained similar between filters (HF1400 1.70 L/h, 95% CI 1.18–2.22 vs. M150 1.67 L/h, 95% CI 1.23–2.11, *P* = 0.915).

### Effect of point of dilution and filter type

#### CVVH

**Fig. 2:**
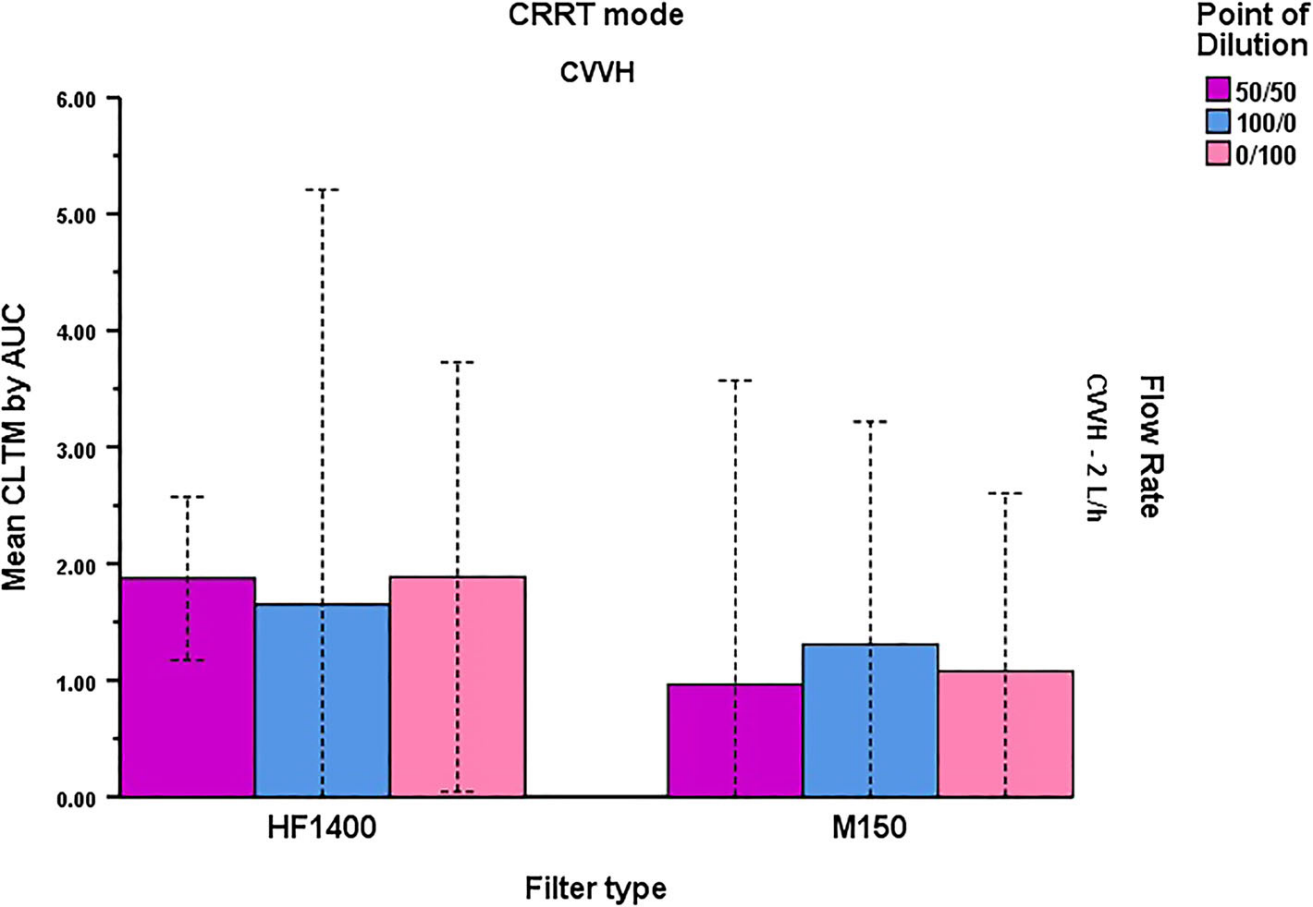
Mean CL_TM_ by AUC of apixaban during CVVH at 2 L/h according to point of dilution and filter type. Bars represent mean values with 95% confidence intervals displayed as dashed lines

These results were confirmed via a two-way ANOVA which demonstrated that the interaction between point of dilution and filter type was not significant on CL_TM_ by AUC (*P* = 0.292) or by SC (*P =* 0.519) at 2 L/h. An analysis of the main effects of each variable indicated that there was a significant difference in CL_TM_ by AUC according to filter type (*P* = 0.003) but not point of dilution (*P* = 0.922), as previously described (Fig. 2). Conversely, CL_TM_ by SC again differed significantly by point of dilution (*P* < 0.001) but not by filter type (*P* = 0.304). As only one point of dilution (50/50%) was tested at the 4 L/h rate (2 experiments), two-way ANOVAs were not performed. Ignoring flow rate generated similar results with a non-significant interaction between filter type and point of dilution for CL_TM_ by AUC (*P =* 0.648) and by SC (*P* = 0.792) while main effects continued to differ according to filter type for CL_TM_ by AUC (*P* = 0.049) but not point of dilution (*P* = 0.238) and vice versa for CL_TM_ by SC (*P* = 0.904 for filter type and *P* < 0.001 for dilution).

#### CVVHD

Similarly for CVVHD at 2 L/h, there was a significant difference in mean CL_TM_ by AUC based on filter type (HF1400 1.67 L/h, 95% CI 0.08–3.25 vs. M150 0.91 L/h, 95% CI 0.46–1.35, *P* = 0.028). This difference remained at 4 L/h with mean CL_TM_ by AUC values of 2.17 L/h (95% CI 0.96–3.37) for HF1400 and 1.63 L/h (95% CI 0.99–2.27) for M150 (*P* = 0.038). For CL_TM_ by SA, mean values did not differ at 2 L/h (HF1400 1.25 L/h, 95% CI 0.93–1.56 vs. M150 1.24 L/h, 95% CI 0.73–1.75, *P* = 0.925) or 4 L/h (HF1400 2.41 L/h, 95% CI 1.71–3.10 vs. M150 2.23 L/h, 2.16–2.29, *P* = 0.083). Ignoring flow rate, mean CL_TM_ by AUC values remained significantly higher for HF1400 (1.92 L/h, 95% CI 1.41–2.42) compared to the M150 (1.27 L/h, 95% CI 0.60–1.94, *P* = 0.049) but did not differ significantly for CL_TM_ by SA (HF1400 1.83 L/h, 95% CI 0.76–2.89 vs. M150 1.73 L/h, 95% CI 0.83–2.64, *P =* 0.841).

### Effect of CRRT mode, filter type, and flow rate

**Fig. 3:**
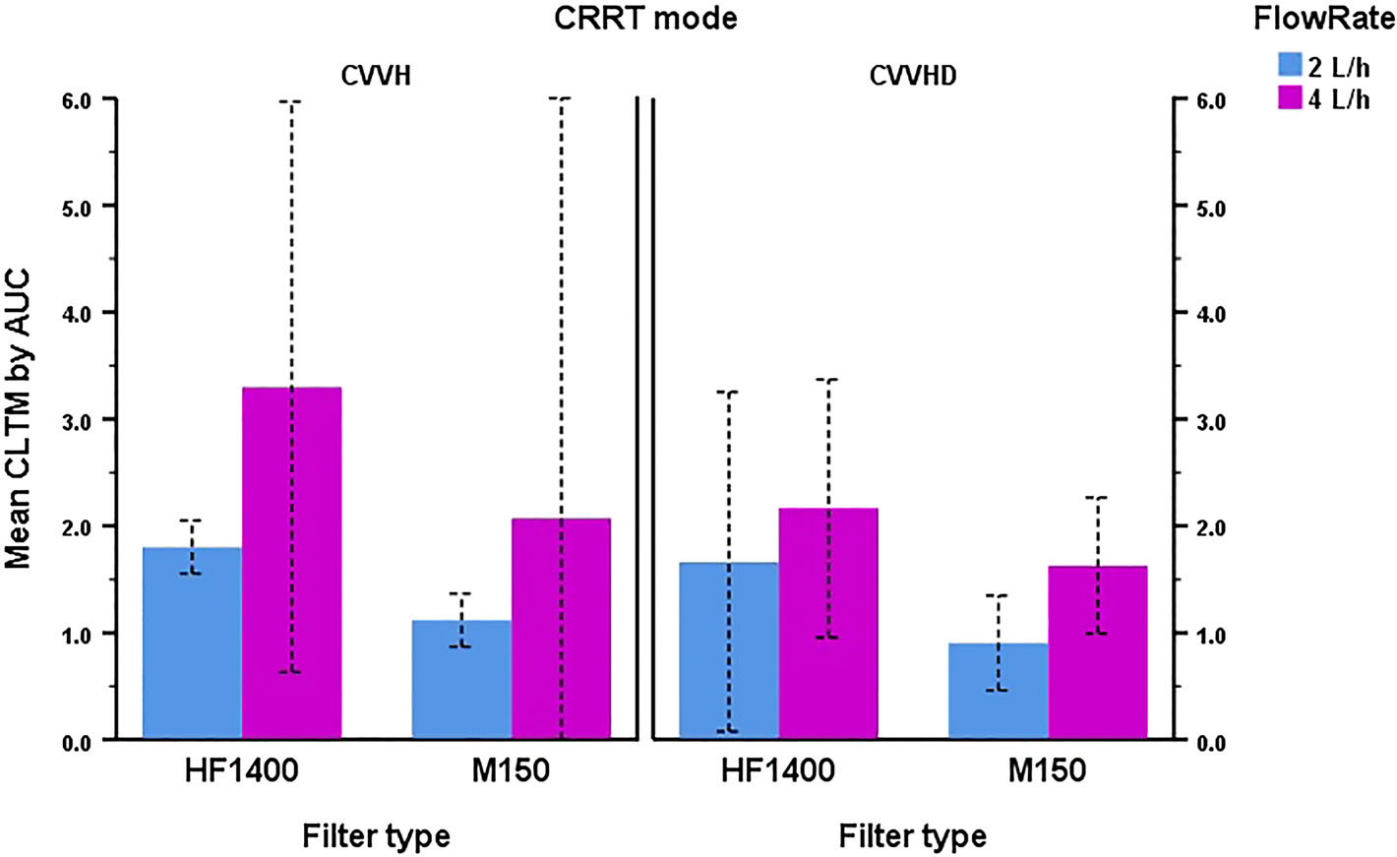
Mean CL_TM_ by AUC of apixaban during in vitro CRRT according to mode, flow rate, and filter type. Bars represent mean values with 95% confidence intervals displayed as dashed lines

Ignoring point of dilution, the three-way ANOVA for the effect of CRRT mode, filter type, and flow rate on CL_TM_ by AUC demonstrated no significant two-way interactions between CRRT mode and filter (*P =* 0.176) or filter and flow rate (*P* = 0.475), while CRRT mode and flow rate was significant at *P* = 0.013 (Fig. 3). The three-way interaction between CRRT mode, filter, and flow rate was also non-significant (*P* = 0.098) with an adjusted R^2^ of 0.869. For the CL_TM_ by SC/SA model, all two-way interactions were non-significant (CRRT mode*filter, *P* = 0.914, filter*flow rate, *P* = 0.427, and CRRT mode*flow rate, *P =* 0.540) along with the three-way interaction (CRRT mode*filter*flow rate, *P* = 0.755, adjusted R^2^ = 0.516). The estimated marginal means for CL_TM_ by AUC and SC/SA generated from these ANOVAs as a function of CRRT mode, filter type, and flow rate are displayed in Table 3.

**Table 3:** Three-way ANOVA-generated marginal means of CL_TM_ by CRRT mode, filter type, and flow rate

CL_TM_ by SC or SA					
CRRT mode	Filter type	Flow rate (L/h)	CL_TM_ (L/h)	95% CI	SE
CVVH	HF1400	2	1.438	1.105–1.772	0.157
		4	2.490	1.913–3.067	0.272
	M150	2	1.507	1.173–1.840	0.157
		4	2.160	1.583–2.737	0.272
CVVHD	HF1400	2	1.245	0.668–1.822	1.245
		4	2.405	1.828–2.982	2.405
	M150	2	1.240	0.663–1.817	0.272
		4	2.225	1.648–2.802	0.272
CL_TM_ by AUC					
CVVH	HF1400	2	1.803	1.596–2.011	0.980
		4	3.300	2.941–3.659	0.170
	M150	2	1.118	0.911–1.326	0.098
		4	2.070	1.711–2.429	0.170
CVVHD	HF1400	2	1.665	1.306–2.024	0.170
		4	2.165	1.806–2.524	0.170
	M150	2	0.905	0.546–1.264	0.170
		4	1.630	1.271–1.989	0.170

*SE* standard error

### Adsorption experiments

The extent of apixaban adsorbed to the CRRT circuit was evaluated across two duplicate experiments with serial samples collected over a span of 180 min; with each filter type in CVVH mode at a flow rate of 2 L/h with a 50/50% dilution scheme. The intra-assay CV% within the HF1400 filter experiments was 5.55 and 10.05% for the M150 filter. Overall, the mean (±SD) percent adsorption differed significantly between the HF1400 and M150 filter types at 38.1 ± 13.4% and 12.8 ± 11.2% (*P* < 0.001). The concentration-time profiles during these experiments are displayed in Supplementary Fig. 1 in which adsorption peaks at 10 min for both filter types, although the amount adsorbed to the M150 filter is roughly half that of the HF1400. While the rate and magnitude of decline in adsorption to the HF1400 filter was significantly less than that of the M150, there was no clear evidence of filter saturation. Regardless of filter type, adsorption appeared reversible corresponding to decreasing circulating apixaban concentrations.

### Protein binding

The protein binding of apixaban has been previously explored in the serum of rats, dogs, chimpanzees, and humans but never in bovine plasma [58]. Protein binding in these species has ranged from 87% in humans to 96% in rats and varied slightly by concentration. Given its impact on drug clearance during CRRT, the protein binding of apixaban to bovine plasma from 4 contrived plasma samples with a measured bovine albumin concentration of 3.597 mg/dL (Biologic Resources Laboratory, University of Illinois at Chicago, Chicago, IL, USA) was evaluated. Samples were spiked at the human simulated C_max_ and 0.5x C_max_ concentration [47]. Overall, the mean (±SD) percent protein binding in bovine plasma was 70.81 ± 0.01% with an intra-assay CV% of 4.2% at C_max_ and 1.2% at 0.5x C_max_.

### Optimal CRRT dose determination

Given the increased precision of CL_TM_ by AUC compared to SC/SA [59], lack of influence by point of dilution, and improved model fit (adjusted R^2^ 0.87 vs. 0.52), these values were used to generate optimal dosing recommendations generalizable across varying CRRT modalities. All 4 applicable covariates (CRRT mode, filter type, flow rate, and point of dilution) were entered into the multiple linear regression model (Table 4). Filter type and flow rate were significant, and therefore, retained in the final model demonstrating a decrease of 0.821 L/h (95% CI − 1.13 to − 0.509, *P* < 0.001) in CL_TM_ when switching from the HF1400 to M150 filter and an increase of 0.612 L/h (95% CI 0.864–1.585, *P* < 0.001) in CL_TM_ for every 1 L/h increase in flow rate with excellent correlation (adjusted R^2^ = 0.849). This regression equation was then used to make predictions for CL_TM_ and estimate optimal dosing recommendations for apixaban during CRRT across filter types and simulated flow rates from 0.5–5 L/h (Table 5). Although renal clearance (CL_R_) typically only accounts for approximately 27% of the CL_T_ of apixaban [46], mean observed CL_TM_ values account for up to 60% of CL_T_ at the highest flow rates simulated in this model (Table 5). As such, the labeled dose of 5 mg twice (BID) would be appropriate for 60–90% of the simulated flow rates, depending on filter type. Given the direct correlation between apixaban concentrations and its pharmacodynamic activity, along with its well-established safety profile at doses up to 50 mg daily for 3–7 days (AUC up to 6045 ng · h/mL) [33, 46], we feel these doses are appropriate to maximize the efficacy and safety of apixaban and match the desired systemic exposure in patients with increased CL_T_ due to high CRRT flow rates. Maintaining single doses ≤10 mg also ensures that its linear PK properties are preserved, and that dissolution-limited absorption and decreases in bioavailability are avoided.

**Table 4:** Multiple linear regression between tested covariates and CL_TM_ by AUC

Variable	Unstandardized β	95% CI	*P* value
Constant	1.469	0.800–2.137	< 0.001
Filter type^a^	−0.821	−1.133 - -0.509	< 0.001
Flow rate (L/h)	0.612	0.864–1.585	< 0.001

^a^HF1400 = 1, M150 = 2

Variables entered: CRRT mode, filter type, flow rate, and point of dilution

Adjusted R^2^ = 0.849

Regression equation: CL_TM_ = 1.469 L/h + (− 0.821 * filter type) + (0.612 * flow rate (L/h))

**Table 5:** Optimal dosing recommendations of apixaban by filter type and CRRT flow rate

	Mean CL_TM_ (L/h)			Mean CL_T_ (L/h)		Optimal total daily dose (mg)^c^			
CRRT flow rate (L/h)	HF1400 Filter	M150 Filter	Mean CL_NR_ (L/h)^a^	HF1400 Filter	M150 Filter	Target AUC (ng · h/mL)^b^	HF1400 Filter	M150 Filter	Optimal dosing regimen^d^
0.5	0.95	0.13	2.52	3.47	2.65	2103.8	7.31	5.58	2.5 mg BID
1	1.26	0.44	2.52	3.78	2.96	2103.8	7.95	6.23	2.5–5 mg BID
1.5	1.57	0.75	2.52	4.09	3.27	2103.8	8.60	6.87	2.5–5 mg BID
2	1.87	1.05	2.52	4.39	3.57	2103.8	9.24	7.51	5 mg BID
2.5	2.18	1.36	2.52	4.70	3.88	2103.8	9.88	8.16	5 mg BID
3	2.48	1.66	2.52	5.00	4.18	2103.8	10.53	8.80	5 mg BID
3.5	2.79	1.97	2.52	5.31	4.49	2103.8	11.17	9.44	5 mg BID
4	3.10	2.28	2.52	5.62	4.80	2103.8	11.82	10.09	5 mg BID
4.5	3.40	2.58	2.52	5.92	5.10	2103.8	12.46	10.73	5 mg BID
5	3.71	2.89	2.52	6.23	5.41	2103.8	13.10	11.38	5–7.5 mg BID

^a^Estimated CL_NR_ from Byon et al. *CPT Pharmacometrics Syst Pharmacol* 2017;6(5):340–349

^b^Target AUC from Frost et al. *Br J Clin Pharmacol* 2013;76:776–786

^c^To achieve comparable mean AUC values achieved in 6 patients administered apixaban 5 mg BID for 7 days, the observed AUC_0–12_ (1051.9 ng · h/mL) was multiplied by two to estimate AUC_0–24_ and solve for dose via the equation AUC = total daily dose/CL_T_

^d^Rounded to the nearest 2.5 mg tablet

## Discussion

To our knowledge this is the first study to evaluate the CL_TM_ of apixaban during CRRT and provides the first set of PK information for which to guide optimal dosing. Our data demonstrate that although it is considered highly protein bound (87%) and minimally renally excreted (27%), the estimated removal of apixaban during CRRT accounted for up to 60% of total clearance and necessitated doses at or above the standard labeled dosage to achieve target therapeutic AUC values [31]. These results underscore the need to thoroughly evaluate the extracorporeal removal of drugs in tightly controlled, rigorous in vitro settings rather than estimating the potential removal using drug- and/or CRRT-specific factors, which has shown to be misleading in previous investigations [60], or by extrapolating from data generated from patients on intermittent hemodialysis [33, 61]. This is especially true as the use of apixaban for therapeutic anticoagulation continues to increase among critically ill ICU patients [8, 62–64], many if not most of whom will require CRRT at some point during their hospitalization.

In addition to providing clinicians with the first set of data for which to guide dosing of apixaban during CRRT, our study has several other notable strengths. Primarily, our methodology for assessing true CL_TM_ employed a rich PK sampling scheme and thorough statistical analyses which significantly improved our ability to accurately estimate drug removal during CRRT. The majority of previous studies employ a single sample design and attempt to estimate CL_TM_ by multiplying SC or SA derived from a single time point by the flow rate [65–68]. These methods falsely assume SC and SA are static over time and that CL_TM_ is directly proportional to flow rate across the continuum of CRRT settings. Moreover, the methods used for calculating SC, SA, and CL_TM_ have varied dramatically throughout the literature, even among the same authors/groups across different studies [50, 69–75], especially with regards to the influence of point of dilution during CVVH (Supplementary Fig. 2). Therefore, our optimal dosing regimens were generated exclusively via noncompartmental analyses given its increased precision, lack of influence by point of dilution, and improved model fit. Additionally, point of dilution was the only covariate not retained in our final multiple linear regression (Table 4), further underscoring its lack of impact.

Finally, we also assessed the effect of protein binding and adsorption on the clearance of apixaban during CRRT. Although protein binding is known to be one of the most important factors affecting drug removal during CRRT [76], exceedingly few agents have available data regarding binding to bovine plasma as these animals are not typically utilized in the drug development process [77]. As the measured protein binding of apixaban in bovine plasma in our study was within 20% of that observed in healthy volunteers, it is unlikely that this significantly altered our optimal dosing recommendations. While crystalloid solutions are often used as the vehicle for drug delivery during in vitro CRRT studies due to accessibility and low cost [53, 78, 79], these solutions lack blood proteins vital for facilitating drug-protein binding and do not allow for the formation of a protein or fibrin layer on the extracorporeal circuit and hemodialyzer membrane. Albeit the use of modern, highly biocompatible hemofilters has often made drug adsorption negligible compared to the effect of filtration, it is critical to evaluate this component of removal from the circuit especially for moderately water soluble, lipophilic drugs like apixaban. Given that peak adsorption almost always occurs within the first 5–30 min of CRRT [66, 80, 81], our 180 min experiments allowed ample time for the deposition of blood proteins to the hemofilter and circuit in order to assess the reversibility or saturation point of apixaban adsorption [82]. We observed degrees of filter adsorption high enough to potentially effect drug dosing during CRRT in addition to filtration, particularly for the HF1400 filter, which necessitated filter-specific dosing recommendations as displayed in Table 5. This observed diparity between the two filter types is likely due to differences in their composition, as previously described [66, 81, 83].

Despite these strengths, our study is not without limitations. First, although as many different CRRT machines, filter types, dilution points, and flow rates as possible were included, the results may not be representative of all modalities of CRRT. Second, we assumed non-renal drug clearance to be stable when estimating dosing recommendations. Although there are some data to suggest AKI may affect non-renal clearance [84], there are currently no practical methods or useful biomarkers to assess changes in non-renal clearance. Third, the lower protein binding of apixaban in bovine plasma compared to humans may have led to increased CRRT clearance and subsequently increased dosing recommendation, although hypoalbuminemia is a common phenomenon among ICU patients undergoing CRRT [85]. Fourth, as mentioned, the necessity of forming a closed system may falsely inflate the degree of filter adsorption observed in vitro as virtually all of the drug in the blood continuously encounters the hemofilter and CRRT circuit, therefore precipitating the maximal degree of drug-filter interaction and adsorptive loss [66]. In addition, the utilization of a new hemofilter and circuit for each experiment may have limited our ability to fully assess the effect of filter life on adsorption and CL_TM_ as well as the impact of repeated apixaban dosing. Due to the complicated logistics of in vitro adsorption experiments, caution should be taken when interpreting these data [86]. Lastly, as the PK of apixaban has already been extensively described [56], our 1-h PK sampling scheme in this study was designed solely to evaluate the CL_TM_ of apixaban during CRRT, and therefore, the half-lives reported should be interpreted in light of this.

## Conclusion

As recognized by the FDA, NIH, and NIAID, non-clinical PK/PD models play a critical role in designing human dosage regimens and are essential tools for drug development and dose optimization in special populations [43]. This study thoroughly explored the PK and dialytic clearance of apixaban during CRRT during tightly controlled in vitro experimentation. Apixaban was approximately 70% bound to bovine plasma and demonstrated variable adsorption to the HF1400 and M150 filters at approximately 38 and 13%, respectively. Clearance of apixaban during CRRT was most accurately estimated via calculation of CL_TM_ through noncompartmental estimation of AUC, as opposed to utilizing the product of SC/SA and flow rate. For CRRT flow rates most commonly employed in clinical practice [87], the standard labeled 5 mg BID dose of apixaban is predicted to achieve target systemic exposure thresholds. The safety and efficacy of these proposed dosing regimens warrants further investigation in in vivo studies of critically ill patients undergoing CRRT.

## Supplementary Information

**Additional file 1: Supplemental Figure 1.** Central reservoir plasma concentration-time profiles of apixaban within closed-circuit adsorption experiments with each filter type. Mean values are displayed with error bars representing standard deviations.

**Additional file 2: Supplemental Figure 2.** Variations in sieving coefficient (SC) during CVVH according to location of replacement fluid infusion, method used for calculation, and inclusion or exclusion of a dilutional correction factor (CF). Sampling locations are as follows: 1) undiluted Cpre; 2) diluted Cpre; 3) undiluted Cpost; 4) diluted Cpost; 5) Cuf.

## Data Availability

The datasets generated and/or analyzed during the current study are not publicly available due confidentiality agreements with the study sponsor but may be available from the corresponding author on reasonable request.

## Abbreviations

AKI: Acute kidney injury
AN69: Acrylonitrile
ANOVA: Analysis of variance
aPTT: Activated thromboplastin time
AUC: Area under the curve
AUC_0-∞_: Area under the curve from time zero to infinity
AUC_0–24_: Area under the curve from time zero to 24 h
AUC_0-last_: Area under the curve from time zero to last measurable concentration
BID: Twice daily
C_dialysate_: Concentration in the dialysate
C_last_: Last observed plasma concentration
C_max_: Maximum plasma concentration
C_post_: Concentration from the post-filter sampling port
C_pre_: Concentration from the pre-filter sampling port
C_uf_: Concentration in the ultrafiltrate
CI: Confidence interval
CL: Clearance
CL_CVVH_: Continuous veno-venous hemofiltration clearance
CL_CVVHD_: Continuous veno-venous hemodialysis clearance
CL_NR_: Non-renal clearance
CL_R_: Renal clearance
CL_T_: Total body clearance
CL_TM_: Transmembrane clearance
CRRT: Continuous renal replacement therapy
CV: Coefficient of variance
CVVH: Continuous veno-venous hemofiltration
CVVHD: Continuous veno-venous hemodialysis
DOAC: Direct oral anticoagulant
ESRD: End stage renal disease
FDA: Food and Drug Administration
HD: Hemodialysis
ICU: Intensive care unit
K_e_: Elimination rate constant
LMWH: Low molecular weight heparin
NIAID: National Institute of Allergy and Infectious Diseases
NIH: National Institute of Health
NOAF: New-onset atrial fibrillation
PAES: Polyarylethersulfone
PD: Pharmacodynamics
PK: Pharmacokinetics
Q_b_: Blood flow rate
Q_d_: Dialysate flow rate
Q_rep_: Pre-filter replacement fluid rate
Q_uf_: Ultrafiltrate or replacement fluid flow rate
QC: Quality control
RE: Relative error
RRT: Renal replacement therapy
SA: Saturation coefficient
SC: Sieving coefficient
SD: Standard deviation
t_1/2_: Half-life
TDD: Total daily dose
UFH: Unfractionated heparin
V_d_: Volume of distribution
VTE: Venous thromboembolism
