# Supplementary material for: Pharmacokinetics and dialytic clearance of apixaban during in vitro continuous renal replacement therapy
Source: BMC Nephrol. 2021 Jan 30;22:45. doi: 10.1186/s12882-021-02248-7 (PMC7847018; doi:10.1186/s12882-021-02248-7)
Supplement: Supplementary file 1 — Additional file 1: Supplemental Figure 1. Central reservoir plasma concentration-time profiles of apixaban within closed-circuit adsorption experiments with each filter type. Mean values are displayed with error bars representing standard deviations. [file 12882_2021_2248_MOESM1_ESM.docx]

**
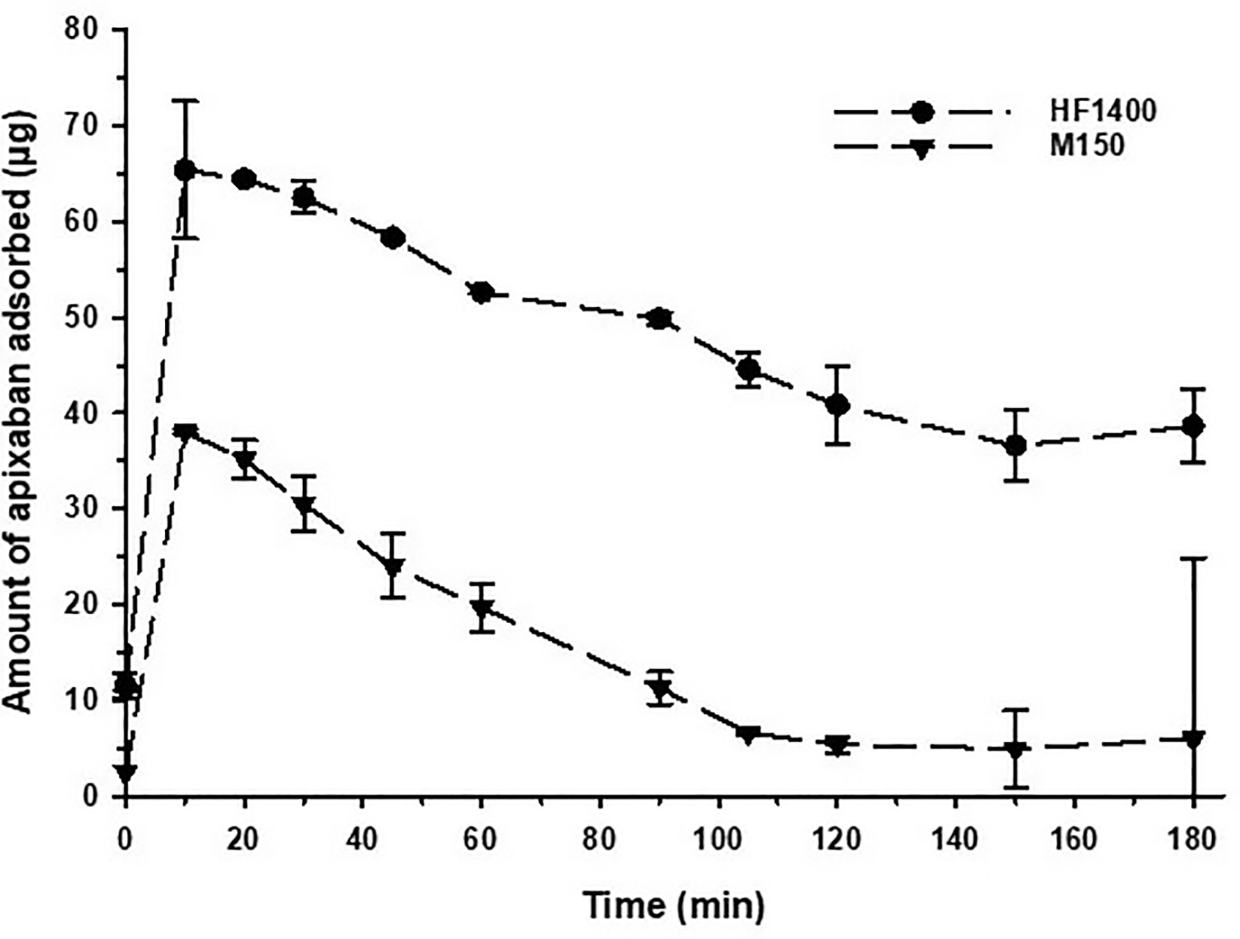
Supplemental Figure 1.** Central reservoir plasma concentration-time profiles of apixaban within closed-circuit adsorption experiments with each filter type. Mean values are displayed with error bars representing standard deviations.
