# Supplementary material for: Pharmacokinetics and dialytic clearance of apixaban during in vitro continuous renal replacement therapy
Source: BMC Nephrol. 2021 Jan 30;22:45. doi: 10.1186/s12882-021-02248-7 (PMC7847018; doi:10.1186/s12882-021-02248-7)

**Supplemental Figure 2.** Variations in sieving coefficient (SC) during CVVH according to location of replacement fluid infusion, method used for calculation, and inclusion or exclusion of a dilutional correction factor (CF). Sampling locations are as follows: 1) undiluted Cpre; 2) diluted Cpre; 3) undiluted Cpost; 4) diluted Cpost; 5) Cuf.


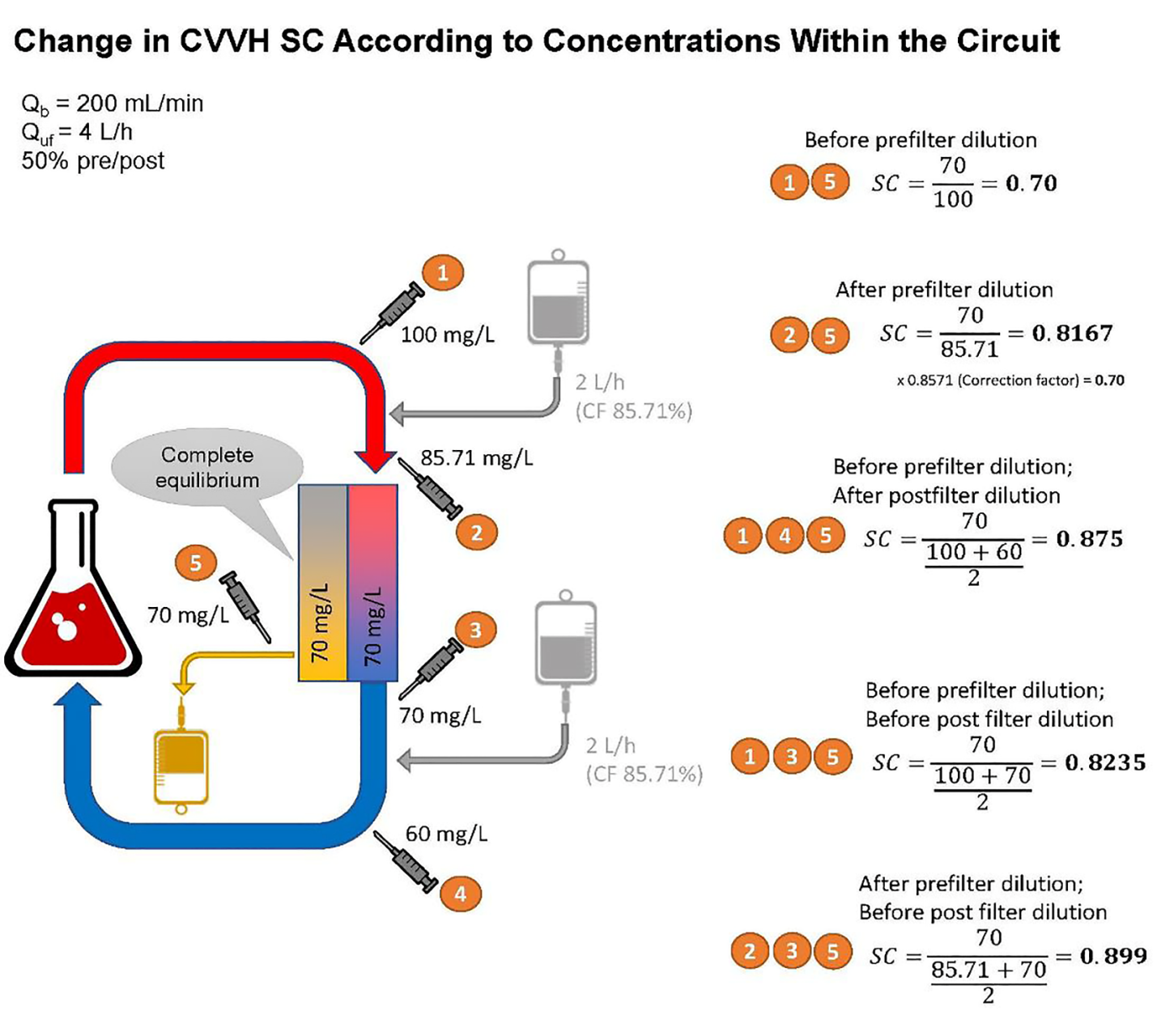

Supplement: Supplementary file 2 — Additional file 2: Supplemental Figure 2. Variations in sieving coefficient (SC) during CVVH according to location of replacement fluid infusion, method used for calculation, and inclusion or exclusion of a dilutional correction factor (CF). Sampling locations are as follows: 1) undiluted Cpre; 2) diluted Cpre; 3) undiluted Cpost; 4) diluted Cpost; 5) Cuf. [file 12882_2021_2248_MOESM2_ESM.docx]
